# Ethyl 2-amino-4-(4-methyl-1,3-thia­zol-5-yl)-5-oxo-4*H*,5*H*-pyrano[3,2-*c*]chromene-3-carboxyl­ate

**DOI:** 10.1107/S1600536813021703

**Published:** 2013-08-10

**Authors:** V. Karthikeyan, V. Ramkumar, R. Joel Karunakaran

**Affiliations:** a Department of Chemistry, Madras Christian College, Tambaram, Chennai 600 059, Tamil Nadu, India; b Department of Chemistry, IIT Madras, Chennai 600 036, Tamil Nadu, India

## Abstract

There are two independent mol­ecules in the asymmetric unit of the title compound, C_19_H_16_N_2_O_5_S, in which the thia­zole rings make dihedral angles of 80.89 (11) and 84.81 (11)° with the pyrano[3,2-*c*]chromene ring systems. An intra­molecular N—H⋯O hydrogen bond involving the amino group occurs in each independent mol­ecule. In the crystal, the amino groups are involved in N—H⋯O and N—H⋯N hydrogen bonds.

## Related literature

Similar conformations were observed in the structures of ethyl 2-amino-5-oxo-4-(*p*-tol­yl)-4*H*,5*H*-pyrano[3,2-*c*]chromene-8-carboxyl­ate (Wang *et al.*, 2004 ▶) and ethyl 2-amino-4-(2,4-di­chloro­phen­yl)-4*H*-benzo[*f*]chromene-3-carboxyl­ate (Shi *et al.*, 2003 ▶). For applications of 4*H*-chromene and its derivatives, see: Jeso & Nicolaou (2009 ▶); Alvey *et al.* (2008 ▶, 2009 ▶); Bedair *et al.* (2001 ▶); El-Agrody *et al.* (2002 ▶, 2011 ▶); Abd-El-Aziz *et al.* (2004 ▶); Sabry *et al.* (2011 ▶). 
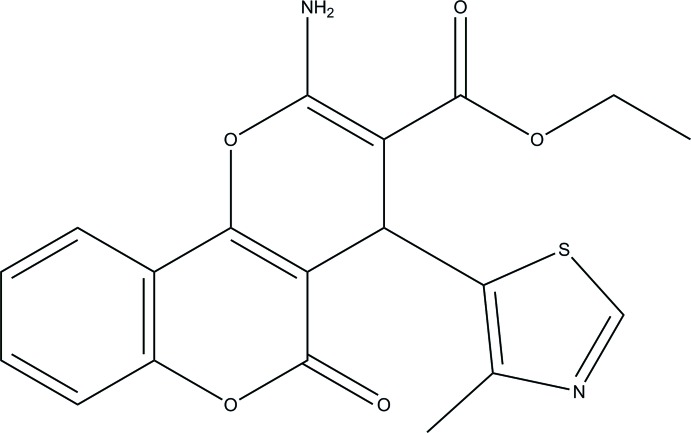


## Experimental

### 

#### Crystal data

C_19_H_16_N_2_O_5_S
*M* *_r_* = 384.41
Monoclinic, 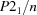

*a* = 15.6232 (10) Å
*b* = 15.0696 (9) Å
*c* = 15.9063 (11) Å
β = 98.873 (2)°
*V* = 3700.1 (4) Å^3^
*Z* = 8
Mo *K*α radiation
μ = 0.21 mm^−1^
*T* = 298 K
0.35 × 0.25 × 0.15 mm

#### Data collection

Bruker APEXII CCD area-detector diffractometer
Absorption correction: multi-scan (*SADABS*; Bruker, 2004 ▶) *T*_min_ = 0.931, *T*_max_ = 0.970
18921 measured reflections
6130 independent reflections
3527 reflections with *I* > 2σ(*I*)
*R*_int_ = 0.042

#### Refinement

*R*[*F* ^2^ > 2σ(*F* ^2^)] = 0.048
*wR*(*F* ^2^) = 0.145
*S* = 1.03
6130 reflections
491 parameters
H-atom parameters constrained
Δρ_max_ = 0.20 e Å^−3^
Δρ_min_ = −0.29 e Å^−3^

### 

Data collection: *APEX2* (Bruker, 2004 ▶); cell refinement: *SAINT-Plus* (Bruker, 2004 ▶); data reduction: *SAINT-Plus*; program(s) used to solve structure: *SHELXS97* (Sheldrick, 2008 ▶); program(s) used to refine structure: *SHELXL97* (Sheldrick, 2008 ▶); molecular graphics: *ORTEP-3 for Windows* (Farrugia, 2012 ▶); software used to prepare material for publication: *SHELXL97*.

## Supplementary Material

Crystal structure: contains datablock(s) global, I. DOI: 10.1107/S1600536813021703/bx2447sup1.cif

Structure factors: contains datablock(s) I. DOI: 10.1107/S1600536813021703/bx2447Isup2.hkl

Supplementary material file. DOI: 10.1107/S1600536813021703/bx2447Isup3.cml

Additional supplementary materials:  crystallographic information; 3D view; checkCIF report

## Figures and Tables

**Table 1:** Hydrogen-bond geometry (Å, °)

*D*—H⋯*A*	*D*—H	H⋯*A*	*D*⋯*A*	*D*—H⋯*A*
N1—H1*B*⋯O4	0.86	2.11	2.698 (3)	125
N1—H1*B*⋯N2*A* ^i^	0.86	2.49	3.152 (3)	135
N1—H1*C*⋯N2^ii^	0.86	2.27	3.116 (4)	167
N1*A*—H1*A*2⋯N2*A* ^iii^	0.86	2.13	2.988 (4)	174
N1*A*—H1*A*1⋯O4*A*	0.86	2.09	2.690 (3)	126
N1*A*—H1*A*1⋯O4^ii^	0.86	2.52	2.936 (3)	111

Symmetry codes: (i) 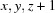; (ii) 
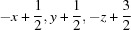
; (iii) 
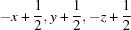
.

**Fig. 1.**
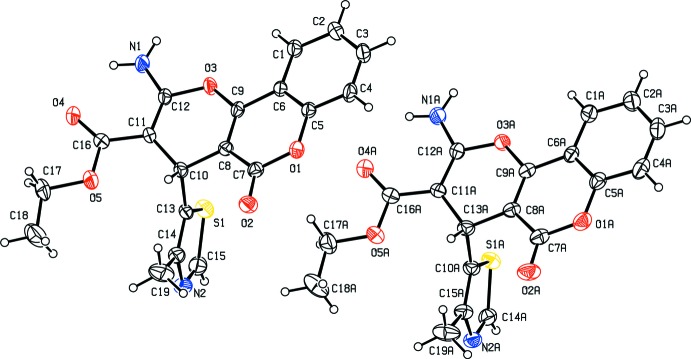
ORTEP of the molecule with atoms represented as 30% probability ellipsoids.

## supplementary crystallographic information

### 1. Comment

Thiazoles are important class of heterocyclic compounds, found in many potent biologically active molecules such as Sulfathiazol (antimicrobial drug), Ritonavir (antiretroviral drug) and Tiazofurin (antineoplastic drug) similarly 4-Hydroxycoumarin forms the nucleus of many natural products and drugs and is also a key intermediate for the widely used oral anticoagulants and rodenticides. There is interest in fused pyranochromenes because chromene derivatives (Jeso & Nicolaou, 2009; Alvey *et al.*, 2008, 2009); can be used as immunomodulators and for the treatment of different diseases of connective tissues, diabetes, anti-cancer and for applications of 4*H*-chromene and its derivatives, (Bedair *et al.* (2001); El-Agrody *et al.* (2002, 2011); Abd-El-Aziz *et al.* (2004); Sabry *et al.* (2011)). We present here the synthesis and crystal structure of the title compound,(I). The molecule is being assessed for biological activity. In the title compound,C_19_H_16_N_2_O_5_, there are two independent molecules in the asymmetric unit. The thiazole rings make a dihedral angle with 4H, 5*H*-pyrano[3,2-*c*]chromene ring system of 80.89 (11) and 84.81 (11)°. In the crystal structure, the amino groups are involved in both intra-and intermolecular N—H···O and N—H···N hydrogen bonds respectively, table1.

### 2. Experimental

A solution of heteroaromatic aldehyde (1 mmol), 1.2 mmol of alkylnitriles, 4-Hydroxy coumarins(1.1 mmol) and catalytic amount of silver trifluoromethanesulfonate (5 mol %) in 10 mL of ethanol and the reaction mixture refluxed for two hours. After completion of the reaction, which was monitored by TLC, the reaction mixture was cooled to room temperature and kept overnight in a refrigerator. The solid mass separated out was filtered and purified by column chromatography using n-Hexane: Ethylacetate mixture in the ratio (3:2). The crude product formed was recrystallized in ethanol.

### 3. Refinement

All hydrogen atoms were fixed geometrically and allowed to ride on the parent carbon atoms with aromatic C—H = 0.93 Å, methine C—H = 0.98 Å me thylene C—H = 0.97 Å and methyl C—H = 0.96 Å. The displacement parameters were set for phenyl H atoms at *U*_iso_(H) = 1.2*U*_eq_(C,N) and for methine,methylene and methyl H atoms at *U*_iso_(H) =1.5*U*_eq_(C).

### Crystal data

**Table:**

C_19_H_16_N_2_O_5_S	*F*(000) = 1600
*M**_r_* = 384.41	*D*_x_ = 1.380 Mg m^−^^3^
Monoclinic, *P*2_1_/*n*	Mo *K*α radiation, λ = 0.71073 Å
*a* = 15.6232 (10) Å	Cell parameters from 3286 reflections
*b* = 15.0696 (9) Å	θ = 2.4–21.4°
*c* = 15.9063 (11) Å	µ = 0.21 mm^−^^1^
β = 98.873 (2)°	*T* = 298 K
*V* = 3700.1 (4) Å^3^	Rectangular, yellow
*Z* = 8	0.35 × 0.25 × 0.15 mm

### Data collection

**Table:**

Bruker APEXII CCD area-detector diffractometer	3527 reflections with *I* > 2σ(*I*)
Radiation source: fine-focus sealed tube	*R*_int_ = 0.042
phi and ω scans	θ_max_ = 25.0°, θ_min_ = 1.9°
Absorption correction: multi-scan (*SADABS*; Bruker, 2004)	*h* = −13→18
*T*_min_ = 0.931, *T*_max_ = 0.970	*k* = −16→14
18921 measured reflections	*l* = −18→18
6130 independent reflections	

### Refinement

**Table:**

Refinement on *F*^2^	0 restraints
Least-squares matrix: full	Hydrogen site location: inferred from neighbouring sites
*R*[*F*^2^ > 2σ(*F*^2^)] = 0.048	H-atom parameters constrained
*wR*(*F*^2^) = 0.145	*w* = 1/[σ^2^(*F*_o_^2^) + (0.0714*P*)^2^] where *P* = (*F*_o_^2^ + 2*F*_c_^2^)/3
*S* = 1.03	(Δ/σ)_max_ = 0.003
6130 reflections	Δρ_max_ = 0.20 e Å^−^^3^
491 parameters	Δρ_min_ = −0.29 e Å^−^^3^

### Special details

**Table:**

Geometry. All e.s.d.'s (except the e.s.d. in the dihedral angle between two l.s. planes) are estimated using the full covariance matrix. The cell e.s.d.'s are taken into account individually in the estimation of e.s.d.'s in distances, angles and torsion angles; correlations between e.s.d.'s in cell parameters are only used when they are defined by crystal symmetry. An approximate (isotropic) treatment of cell e.s.d.'s is used for estimating e.s.d.'s involving l.s. planes.

### Fractional atomic coordinates and isotropic or equivalent isotropic displacement parameters (Å^2^)

**Table:**

	*x*	*y*	*z*	*U*_iso_*/*U*_eq_	
C1	0.3585 (2)	0.4642 (2)	0.70875 (18)	0.0433 (8)	
H1	0.3209	0.4686	0.7485	0.052*	
C2	0.3845 (2)	0.5395 (2)	0.67213 (19)	0.0500 (9)	
H2	0.3639	0.5946	0.6862	0.060*	
C3	0.4417 (2)	0.5334 (2)	0.61380 (19)	0.0510 (9)	
H3	0.4599	0.5847	0.5894	0.061*	
C4	0.4715 (2)	0.4524 (2)	0.59173 (18)	0.0483 (9)	
H4	0.5098	0.4484	0.5526	0.058*	
C5	0.44379 (18)	0.3771 (2)	0.62839 (17)	0.0360 (7)	
C6	0.38746 (18)	0.38107 (19)	0.68747 (16)	0.0332 (7)	
C7	0.45393 (19)	0.2176 (2)	0.63881 (17)	0.0380 (7)	
C8	0.39486 (18)	0.22000 (19)	0.69980 (16)	0.0334 (7)	
C9	0.36581 (18)	0.2982 (2)	0.72308 (16)	0.0343 (7)	
C10	0.37038 (19)	0.13370 (18)	0.73763 (17)	0.0357 (7)	
H10	0.4235	0.0987	0.7523	0.043*	
C11	0.33538 (19)	0.15276 (19)	0.81953 (17)	0.0357 (7)	
C12	0.30737 (19)	0.2347 (2)	0.83743 (17)	0.0374 (7)	
C13	0.30880 (19)	0.08051 (18)	0.67326 (17)	0.0359 (7)	
C14	0.3214 (2)	−0.0002 (2)	0.63837 (19)	0.0453 (8)	
C15	0.1869 (3)	0.0224 (2)	0.5769 (2)	0.0572 (10)	
H15	0.1343	0.0116	0.5422	0.069*	
C16	0.3354 (2)	0.0848 (2)	0.88338 (19)	0.0449 (8)	
C17	0.3666 (4)	−0.0665 (3)	0.9178 (2)	0.1105 (19)	
H17A	0.4128	−0.0588	0.9656	0.133*	
H17B	0.3120	−0.0676	0.9397	0.133*	
C18	0.3781 (3)	−0.1472 (3)	0.8767 (3)	0.1049 (16)	
H18A	0.3376	−0.1507	0.8248	0.157*	
H18B	0.3684	−0.1957	0.9132	0.157*	
H18C	0.4361	−0.1503	0.8639	0.157*	
C19	0.4028 (3)	−0.0539 (2)	0.6518 (2)	0.0752 (12)	
H19A	0.4459	−0.0238	0.6912	0.113*	
H19B	0.4235	−0.0613	0.5985	0.113*	
H19C	0.3912	−0.1110	0.6743	0.113*	
C1A	0.3840 (2)	0.7211 (2)	0.20315 (18)	0.0483 (9)	
H1A	0.3506	0.7324	0.2456	0.058*	
C2A	0.4120 (2)	0.7901 (2)	0.1583 (2)	0.0562 (9)	
H2A	0.3975	0.8481	0.1703	0.067*	
C3A	0.4616 (2)	0.7739 (3)	0.0952 (2)	0.0599 (10)	
H3A	0.4804	0.8212	0.0653	0.072*	
C4A	0.4834 (2)	0.6890 (3)	0.07611 (19)	0.0579 (10)	
H4A	0.5165	0.6783	0.0333	0.069*	
C5A	0.4555 (2)	0.6196 (2)	0.12157 (18)	0.0450 (8)	
C6A	0.40567 (19)	0.6337 (2)	0.18521 (17)	0.0395 (8)	
C7A	0.4552 (2)	0.4608 (2)	0.14276 (19)	0.0480 (9)	
C8A	0.40041 (19)	0.4741 (2)	0.20780 (17)	0.0392 (8)	
C9A	0.37984 (19)	0.5566 (2)	0.22772 (17)	0.0392 (8)	
C10A	0.3687 (2)	0.39270 (19)	0.24977 (18)	0.0415 (8)	
H10A	0.4182	0.3532	0.2665	0.050*	
C11A	0.33376 (19)	0.4208 (2)	0.32967 (17)	0.0382 (8)	
C12A	0.3164 (2)	0.5074 (2)	0.34506 (18)	0.0413 (8)	
C13A	0.30238 (19)	0.34343 (19)	0.18653 (18)	0.0394 (8)	
C14A	0.1765 (2)	0.2990 (2)	0.08913 (18)	0.0489 (9)	
H14A	0.1233	0.2933	0.0542	0.059*	
C15A	0.3095 (2)	0.2642 (2)	0.14837 (19)	0.0449 (8)	
C16A	0.3177 (2)	0.3561 (2)	0.3915 (2)	0.0472 (8)	
C17A	0.3257 (3)	0.2039 (2)	0.4303 (2)	0.0780 (13)	
H17C	0.2641	0.1987	0.4319	0.094*	
H17D	0.3546	0.2182	0.4871	0.094*	
C18A	0.3591 (4)	0.1201 (3)	0.4024 (3)	0.132 (2)	
H18D	0.3315	0.1071	0.3455	0.198*	
H18E	0.3471	0.0733	0.4399	0.198*	
H18F	0.4205	0.1248	0.4034	0.198*	
C19A	0.3861 (2)	0.2038 (2)	0.1600 (2)	0.0764 (12)	
H20X	0.4044	0.1934	0.1059	0.115*	
H19X	0.3706	0.1484	0.1834	0.115*	
H21X	0.4325	0.2307	0.1981	0.115*	
N1	0.27345 (17)	0.26186 (17)	0.90444 (15)	0.0552 (8)	
H1B	0.2668	0.2250	0.9442	0.066*	
H1C	0.2581	0.3164	0.9082	0.066*	
N2	0.2506 (2)	−0.03345 (19)	0.58422 (16)	0.0541 (7)	
N1A	0.28373 (18)	0.54145 (17)	0.40989 (15)	0.0577 (8)	
H1A1	0.2706	0.5073	0.4493	0.069*	
H1A2	0.2756	0.5978	0.4126	0.069*	
N2A	0.23690 (18)	0.23858 (17)	0.09262 (15)	0.0473 (7)	
O1	0.47619 (13)	0.29715 (14)	0.60465 (11)	0.0430 (5)	
O2	0.48555 (14)	0.15115 (14)	0.61402 (13)	0.0505 (6)	
O3	0.31266 (13)	0.30626 (12)	0.78376 (12)	0.0434 (5)	
O4	0.31017 (17)	0.09227 (14)	0.95117 (14)	0.0642 (7)	
O5	0.36704 (17)	0.00778 (15)	0.85867 (13)	0.0666 (7)	
O1A	0.47929 (14)	0.53504 (16)	0.10116 (12)	0.0537 (6)	
O2A	0.48122 (15)	0.39022 (17)	0.12158 (15)	0.0656 (7)	
O3A	0.33117 (14)	0.57478 (12)	0.29067 (12)	0.0453 (6)	
O4A	0.28704 (17)	0.36983 (14)	0.45668 (14)	0.0619 (7)	
O5A	0.34104 (15)	0.27344 (14)	0.37173 (13)	0.0590 (6)	
S1	0.20650 (6)	0.11782 (6)	0.63551 (5)	0.0507 (3)	
S1A	0.20197 (5)	0.38856 (5)	0.15289 (5)	0.0484 (3)	

### Atomic displacement parameters (Å^2^)

**Table:**

	*U*^11^	*U*^22^	*U*^33^	*U*^12^	*U*^13^	*U*^23^
C1	0.049 (2)	0.042 (2)	0.0415 (18)	−0.0003 (16)	0.0142 (15)	0.0060 (16)
C2	0.060 (2)	0.037 (2)	0.053 (2)	−0.0018 (17)	0.0116 (18)	0.0078 (16)
C3	0.064 (2)	0.044 (2)	0.0453 (19)	−0.0088 (18)	0.0071 (17)	0.0127 (16)
C4	0.057 (2)	0.054 (2)	0.0367 (18)	−0.0094 (19)	0.0154 (16)	0.0099 (16)
C5	0.0385 (18)	0.038 (2)	0.0321 (16)	−0.0052 (15)	0.0058 (14)	0.0017 (14)
C6	0.0364 (17)	0.0336 (19)	0.0297 (15)	−0.0022 (15)	0.0054 (13)	0.0035 (13)
C7	0.0371 (18)	0.042 (2)	0.0356 (17)	−0.0046 (16)	0.0073 (14)	−0.0029 (15)
C8	0.0348 (18)	0.0332 (19)	0.0331 (16)	−0.0007 (14)	0.0086 (14)	−0.0013 (13)
C9	0.0341 (18)	0.042 (2)	0.0287 (15)	−0.0054 (15)	0.0091 (13)	0.0040 (14)
C10	0.0395 (18)	0.0319 (18)	0.0375 (16)	−0.0021 (14)	0.0117 (14)	0.0029 (13)
C11	0.0462 (19)	0.0272 (19)	0.0343 (16)	−0.0032 (15)	0.0087 (14)	0.0055 (13)
C12	0.0440 (19)	0.036 (2)	0.0338 (16)	−0.0011 (15)	0.0126 (14)	0.0060 (14)
C13	0.0435 (19)	0.0272 (18)	0.0389 (17)	−0.0025 (14)	0.0122 (14)	0.0020 (13)
C14	0.054 (2)	0.040 (2)	0.0455 (19)	−0.0012 (17)	0.0175 (17)	−0.0026 (15)
C15	0.064 (3)	0.059 (3)	0.047 (2)	−0.018 (2)	0.0033 (18)	−0.0006 (17)
C16	0.062 (2)	0.033 (2)	0.0415 (19)	−0.0010 (17)	0.0118 (17)	−0.0003 (16)
C17	0.229 (6)	0.041 (3)	0.066 (3)	0.024 (3)	0.037 (3)	0.024 (2)
C18	0.152 (5)	0.048 (3)	0.103 (3)	0.003 (3)	−0.018 (3)	0.014 (2)
C19	0.078 (3)	0.054 (3)	0.097 (3)	0.011 (2)	0.024 (2)	−0.020 (2)
C1A	0.055 (2)	0.048 (2)	0.0406 (18)	−0.0030 (18)	0.0042 (16)	0.0028 (16)
C2A	0.060 (2)	0.052 (2)	0.053 (2)	−0.0077 (19)	−0.0011 (19)	0.0086 (18)
C3A	0.066 (3)	0.064 (3)	0.045 (2)	−0.020 (2)	−0.0038 (19)	0.0170 (19)
C4A	0.059 (2)	0.080 (3)	0.0356 (18)	−0.016 (2)	0.0104 (16)	0.0028 (19)
C5A	0.044 (2)	0.055 (2)	0.0348 (17)	−0.0073 (18)	0.0022 (15)	−0.0017 (16)
C6A	0.0421 (19)	0.047 (2)	0.0291 (16)	−0.0042 (16)	0.0041 (14)	0.0013 (14)
C7A	0.045 (2)	0.057 (3)	0.0427 (19)	−0.0045 (19)	0.0114 (16)	−0.0123 (18)
C8A	0.0362 (19)	0.044 (2)	0.0379 (17)	−0.0043 (15)	0.0094 (14)	−0.0087 (15)
C9A	0.0403 (19)	0.045 (2)	0.0329 (17)	−0.0008 (16)	0.0064 (14)	−0.0064 (15)
C10A	0.0434 (19)	0.038 (2)	0.0440 (18)	0.0058 (16)	0.0110 (15)	−0.0054 (15)
C11A	0.045 (2)	0.031 (2)	0.0392 (17)	−0.0036 (15)	0.0090 (15)	−0.0037 (14)
C12A	0.048 (2)	0.041 (2)	0.0375 (18)	−0.0030 (16)	0.0149 (15)	0.0013 (15)
C13A	0.042 (2)	0.0343 (19)	0.0441 (18)	0.0028 (15)	0.0128 (15)	−0.0048 (15)
C14A	0.052 (2)	0.053 (2)	0.0424 (19)	−0.0091 (19)	0.0088 (16)	−0.0053 (16)
C15A	0.047 (2)	0.039 (2)	0.0494 (19)	0.0021 (17)	0.0112 (17)	−0.0092 (16)
C16A	0.056 (2)	0.037 (2)	0.048 (2)	−0.0064 (17)	0.0073 (17)	−0.0043 (16)
C17A	0.114 (4)	0.051 (3)	0.060 (2)	−0.009 (2)	−0.014 (2)	0.015 (2)
C18A	0.224 (7)	0.041 (3)	0.134 (4)	0.026 (3)	0.038 (4)	0.008 (3)
C19A	0.070 (3)	0.059 (3)	0.097 (3)	0.019 (2)	0.002 (2)	−0.029 (2)
N1	0.088 (2)	0.0408 (17)	0.0458 (16)	0.0083 (15)	0.0388 (16)	0.0078 (13)
N2	0.066 (2)	0.0483 (19)	0.0489 (17)	−0.0119 (17)	0.0105 (15)	−0.0081 (14)
N1A	0.090 (2)	0.0409 (17)	0.0509 (17)	0.0079 (15)	0.0370 (16)	0.0016 (13)
N2A	0.0552 (19)	0.0448 (18)	0.0449 (16)	−0.0033 (15)	0.0168 (14)	−0.0096 (13)
O1	0.0501 (14)	0.0427 (14)	0.0400 (12)	−0.0067 (11)	0.0194 (10)	0.0034 (10)
O2	0.0545 (15)	0.0446 (15)	0.0574 (14)	0.0001 (12)	0.0247 (12)	−0.0051 (11)
O3	0.0574 (14)	0.0333 (12)	0.0454 (12)	0.0046 (10)	0.0272 (11)	0.0093 (10)
O4	0.110 (2)	0.0454 (14)	0.0434 (13)	−0.0031 (14)	0.0307 (14)	0.0088 (11)
O5	0.114 (2)	0.0379 (15)	0.0519 (14)	0.0119 (14)	0.0255 (14)	0.0121 (11)
O1A	0.0576 (15)	0.0633 (17)	0.0442 (13)	−0.0056 (13)	0.0208 (11)	−0.0059 (12)
O2A	0.0713 (18)	0.0610 (17)	0.0717 (16)	0.0015 (14)	0.0345 (14)	−0.0232 (13)
O3A	0.0601 (15)	0.0358 (13)	0.0453 (12)	0.0057 (11)	0.0251 (11)	0.0003 (10)
O4A	0.0930 (19)	0.0478 (15)	0.0487 (14)	−0.0059 (13)	0.0231 (13)	0.0018 (11)
O5A	0.0852 (19)	0.0352 (15)	0.0562 (14)	0.0033 (13)	0.0094 (13)	0.0022 (11)
S1	0.0503 (6)	0.0434 (6)	0.0562 (5)	−0.0018 (4)	0.0015 (4)	0.0026 (4)
S1A	0.0482 (5)	0.0384 (5)	0.0594 (5)	0.0043 (4)	0.0107 (4)	−0.0031 (4)

### Geometric parameters (Å, º)

**Table:**

C1—C2	1.365 (4)	C1A—H1A	0.9300
C1—C6	1.392 (4)	C2A—C3A	1.380 (4)
C1—H1	0.9300	C2A—H2A	0.9300
C2—C3	1.387 (4)	C3A—C4A	1.370 (5)
C2—H2	0.9300	C3A—H3A	0.9300
C3—C4	1.372 (4)	C4A—C5A	1.380 (4)
C3—H3	0.9300	C4A—H4A	0.9300
C4—C5	1.375 (4)	C5A—O1A	1.380 (4)
C4—H4	0.9300	C5A—C6A	1.385 (4)
C5—O1	1.382 (3)	C6A—C9A	1.433 (4)
C5—C6	1.384 (4)	C7A—O2A	1.205 (4)
C6—C9	1.433 (4)	C7A—O1A	1.381 (4)
C7—O2	1.209 (3)	C7A—C8A	1.455 (4)
C7—O1	1.383 (3)	C8A—C9A	1.335 (4)
C7—C8	1.439 (4)	C8A—C10A	1.516 (4)
C8—C9	1.336 (4)	C9A—O3A	1.375 (3)
C8—C10	1.507 (4)	C10A—C11A	1.518 (4)
C9—O3	1.372 (3)	C10A—C13A	1.521 (4)
C10—C11	1.516 (4)	C10A—H10A	0.9800
C10—C13	1.521 (4)	C11A—C12A	1.363 (4)
C10—H10	0.9800	C11A—C16A	1.434 (4)
C11—C12	1.355 (4)	C12A—N1A	1.323 (3)
C11—C16	1.442 (4)	C12A—O3A	1.376 (3)
C12—N1	1.326 (3)	C13A—C15A	1.352 (4)
C12—O3	1.385 (3)	C13A—S1A	1.718 (3)
C13—C14	1.364 (4)	C14A—N2A	1.305 (4)
C13—S1	1.713 (3)	C14A—S1A	1.699 (3)
C14—N2	1.387 (4)	C14A—H14A	0.9300
C14—C19	1.494 (5)	C15A—N2A	1.382 (4)
C15—N2	1.294 (4)	C15A—C19A	1.492 (4)
C15—S1	1.715 (3)	C16A—O4A	1.225 (3)
C15—H15	0.9300	C16A—O5A	1.349 (4)
C16—O4	1.209 (3)	C17A—O5A	1.446 (4)
C16—O5	1.344 (4)	C17A—C18A	1.461 (5)
C17—C18	1.406 (5)	C17A—H17C	0.9700
C17—O5	1.463 (4)	C17A—H17D	0.9700
C17—H17A	0.9700	C18A—H18D	0.9600
C17—H17B	0.9700	C18A—H18E	0.9600
C18—H18A	0.9600	C18A—H18F	0.9600
C18—H18B	0.9600	C19A—H20X	0.9600
C18—H18C	0.9600	C19A—H19X	0.9600
C19—H19A	0.9600	C19A—H21X	0.9600
C19—H19B	0.9600	N1—H1B	0.8600
C19—H19C	0.9600	N1—H1C	0.8600
C1A—C2A	1.371 (4)	N1A—H1A1	0.8600
C1A—C6A	1.401 (4)	N1A—H1A2	0.8600
			
C2—C1—C6	121.0 (3)	C2A—C3A—H3A	119.5
C2—C1—H1	119.5	C3A—C4A—C5A	118.8 (3)
C6—C1—H1	119.5	C3A—C4A—H4A	120.6
C1—C2—C3	119.7 (3)	C5A—C4A—H4A	120.6
C1—C2—H2	120.2	O1A—C5A—C4A	117.2 (3)
C3—C2—H2	120.2	O1A—C5A—C6A	121.1 (3)
C4—C3—C2	120.6 (3)	C4A—C5A—C6A	121.7 (3)
C4—C3—H3	119.7	C5A—C6A—C1A	118.3 (3)
C2—C3—H3	119.7	C5A—C6A—C9A	116.8 (3)
C3—C4—C5	118.9 (3)	C1A—C6A—C9A	124.9 (3)
C3—C4—H4	120.5	O2A—C7A—O1A	116.9 (3)
C5—C4—H4	120.5	O2A—C7A—C8A	125.5 (3)
C4—C5—O1	116.8 (3)	O1A—C7A—C8A	117.6 (3)
C4—C5—C6	121.8 (3)	C9A—C8A—C7A	119.1 (3)
O1—C5—C6	121.4 (3)	C9A—C8A—C10A	122.8 (3)
C5—C6—C1	117.9 (3)	C7A—C8A—C10A	118.1 (3)
C5—C6—C9	116.4 (3)	C8A—C9A—O3A	122.6 (3)
C1—C6—C9	125.7 (3)	C8A—C9A—C6A	123.2 (3)
O2—C7—O1	116.7 (3)	O3A—C9A—C6A	114.2 (3)
O2—C7—C8	125.3 (3)	C8A—C10A—C11A	109.1 (2)
O1—C7—C8	118.0 (3)	C8A—C10A—C13A	109.8 (2)
C9—C8—C7	119.3 (3)	C11A—C10A—C13A	113.2 (2)
C9—C8—C10	122.2 (2)	C8A—C10A—H10A	108.2
C7—C8—C10	118.4 (3)	C11A—C10A—H10A	108.2
C8—C9—O3	122.8 (2)	C13A—C10A—H10A	108.2
C8—C9—C6	123.2 (2)	C12A—C11A—C16A	117.8 (3)
O3—C9—C6	114.0 (2)	C12A—C11A—C10A	121.6 (3)
C8—C10—C11	109.1 (2)	C16A—C11A—C10A	120.5 (3)
C8—C10—C13	111.1 (2)	N1A—C12A—C11A	128.5 (3)
C11—C10—C13	113.8 (2)	N1A—C12A—O3A	109.0 (3)
C8—C10—H10	107.5	C11A—C12A—O3A	122.4 (2)
C11—C10—H10	107.5	C15A—C13A—C10A	129.3 (3)
C13—C10—H10	107.5	C15A—C13A—S1A	110.1 (2)
C12—C11—C16	117.6 (3)	C10A—C13A—S1A	120.6 (2)
C12—C11—C10	121.6 (2)	N2A—C14A—S1A	115.3 (3)
C16—C11—C10	120.7 (3)	N2A—C14A—H14A	122.3
N1—C12—C11	129.2 (3)	S1A—C14A—H14A	122.3
N1—C12—O3	108.9 (3)	C13A—C15A—N2A	114.7 (3)
C11—C12—O3	121.9 (2)	C13A—C15A—C19A	127.1 (3)
C14—C13—C10	128.9 (3)	N2A—C15A—C19A	118.2 (3)
C14—C13—S1	109.6 (2)	O4A—C16A—O5A	120.5 (3)
C10—C13—S1	121.5 (2)	O4A—C16A—C11A	126.8 (3)
C13—C14—N2	115.1 (3)	O5A—C16A—C11A	112.7 (3)
C13—C14—C19	126.7 (3)	O5A—C17A—C18A	109.2 (3)
N2—C14—C19	118.2 (3)	O5A—C17A—H17C	109.8
N2—C15—S1	115.2 (3)	C18A—C17A—H17C	109.8
N2—C15—H15	122.4	O5A—C17A—H17D	109.8
S1—C15—H15	122.4	C18A—C17A—H17D	109.8
O4—C16—O5	121.7 (3)	H17C—C17A—H17D	108.3
O4—C16—C11	126.7 (3)	C17A—C18A—H18D	109.5
O5—C16—C11	111.5 (3)	C17A—C18A—H18E	109.5
C18—C17—O5	110.4 (3)	H18D—C18A—H18E	109.5
C18—C17—H17A	109.6	C17A—C18A—H18F	109.5
O5—C17—H17A	109.6	H18D—C18A—H18F	109.5
C18—C17—H17B	109.6	H18E—C18A—H18F	109.5
O5—C17—H17B	109.6	C15A—C19A—H20X	109.5
H17A—C17—H17B	108.1	C15A—C19A—H19X	109.5
C17—C18—H18A	109.5	H20X—C19A—H19X	109.5
C17—C18—H18B	109.5	C15A—C19A—H21X	109.5
H18A—C18—H18B	109.5	H20X—C19A—H21X	109.5
C17—C18—H18C	109.5	H19X—C19A—H21X	109.5
H18A—C18—H18C	109.5	C12—N1—H1B	120.0
H18B—C18—H18C	109.5	C12—N1—H1C	120.0
C14—C19—H19A	109.5	H1B—N1—H1C	120.0
C14—C19—H19B	109.5	C15—N2—C14	110.4 (3)
H19A—C19—H19B	109.5	C12A—N1A—H1A1	120.0
C14—C19—H19C	109.5	C12A—N1A—H1A2	120.0
H19A—C19—H19C	109.5	H1A1—N1A—H1A2	120.0
H19B—C19—H19C	109.5	C14A—N2A—C15A	110.4 (3)
C2A—C1A—C6A	120.0 (3)	C5—O1—C7	121.6 (2)
C2A—C1A—H1A	120.0	C9—O3—C12	117.7 (2)
C6A—C1A—H1A	120.0	C16—O5—C17	115.7 (3)
C1A—C2A—C3A	120.2 (3)	C5A—O1A—C7A	122.1 (2)
C1A—C2A—H2A	119.9	C9A—O3A—C12A	118.4 (2)
C3A—C2A—H2A	119.9	C16A—O5A—C17A	116.2 (3)
C4A—C3A—C2A	121.0 (3)	C13—S1—C15	89.65 (17)
C4A—C3A—H3A	119.5	C14A—S1A—C13A	89.39 (16)
			
C6—C1—C2—C3	−1.0 (5)	C7A—C8A—C9A—C6A	3.1 (5)
C1—C2—C3—C4	0.8 (5)	C10A—C8A—C9A—C6A	−176.5 (3)
C2—C3—C4—C5	0.0 (5)	C5A—C6A—C9A—C8A	−1.0 (5)
C3—C4—C5—O1	−179.5 (3)	C1A—C6A—C9A—C8A	178.7 (3)
C3—C4—C5—C6	−0.7 (5)	C5A—C6A—C9A—O3A	179.5 (3)
C4—C5—C6—C1	0.5 (4)	C1A—C6A—C9A—O3A	−0.8 (4)
O1—C5—C6—C1	179.3 (2)	C9A—C8A—C10A—C11A	−15.1 (4)
C4—C5—C6—C9	−178.0 (3)	C7A—C8A—C10A—C11A	165.2 (3)
O1—C5—C6—C9	0.8 (4)	C9A—C8A—C10A—C13A	109.5 (3)
C2—C1—C6—C5	0.3 (5)	C7A—C8A—C10A—C13A	−70.2 (3)
C2—C1—C6—C9	178.7 (3)	C8A—C10A—C11A—C12A	14.3 (4)
O2—C7—C8—C9	178.7 (3)	C13A—C10A—C11A—C12A	−108.3 (3)
O1—C7—C8—C9	−2.0 (4)	C8A—C10A—C11A—C16A	−165.9 (3)
O2—C7—C8—C10	0.9 (5)	C13A—C10A—C11A—C16A	71.4 (4)
O1—C7—C8—C10	−179.8 (2)	C16A—C11A—C12A—N1A	−1.9 (5)
C7—C8—C9—O3	−176.8 (2)	C10A—C11A—C12A—N1A	177.8 (3)
C10—C8—C9—O3	0.9 (4)	C16A—C11A—C12A—O3A	178.7 (3)
C7—C8—C9—C6	2.9 (4)	C10A—C11A—C12A—O3A	−1.6 (5)
C10—C8—C9—C6	−179.4 (3)	C8A—C10A—C13A—C15A	109.8 (3)
C5—C6—C9—C8	−2.3 (4)	C11A—C10A—C13A—C15A	−128.0 (3)
C1—C6—C9—C8	179.4 (3)	C8A—C10A—C13A—S1A	−69.4 (3)
C5—C6—C9—O3	177.5 (2)	C11A—C10A—C13A—S1A	52.8 (3)
C1—C6—C9—O3	−0.9 (4)	C10A—C13A—C15A—N2A	−179.1 (3)
C9—C8—C10—C11	−17.5 (4)	S1A—C13A—C15A—N2A	0.2 (3)
C7—C8—C10—C11	160.3 (3)	C10A—C13A—C15A—C19A	0.6 (5)
C9—C8—C10—C13	108.7 (3)	S1A—C13A—C15A—C19A	179.9 (3)
C7—C8—C10—C13	−73.5 (3)	C12A—C11A—C16A—O4A	2.4 (5)
C8—C10—C11—C12	18.3 (4)	C10A—C11A—C16A—O4A	−177.4 (3)
C13—C10—C11—C12	−106.3 (3)	C12A—C11A—C16A—O5A	−177.0 (3)
C8—C10—C11—C16	−158.2 (3)	C10A—C11A—C16A—O5A	3.3 (4)
C13—C10—C11—C16	77.2 (3)	S1—C15—N2—C14	−1.1 (3)
C16—C11—C12—N1	−5.1 (5)	C13—C14—N2—C15	1.7 (4)
C10—C11—C12—N1	178.3 (3)	C19—C14—N2—C15	−177.3 (3)
C16—C11—C12—O3	173.7 (3)	S1A—C14A—N2A—C15A	−0.9 (3)
C10—C11—C12—O3	−2.9 (4)	C13A—C15A—N2A—C14A	0.4 (4)
C8—C10—C13—C14	116.2 (3)	C19A—C15A—N2A—C14A	−179.2 (3)
C11—C10—C13—C14	−120.3 (3)	C4—C5—O1—C7	178.8 (3)
C8—C10—C13—S1	−65.5 (3)	C6—C5—O1—C7	−0.1 (4)
C11—C10—C13—S1	58.1 (3)	O2—C7—O1—C5	180.0 (3)
C10—C13—C14—N2	176.9 (3)	C8—C7—O1—C5	0.6 (4)
S1—C13—C14—N2	−1.6 (3)	C8—C9—O3—C12	16.9 (4)
C10—C13—C14—C19	−4.1 (5)	C6—C9—O3—C12	−162.8 (2)
S1—C13—C14—C19	177.4 (3)	N1—C12—O3—C9	163.3 (2)
C12—C11—C16—O4	2.6 (5)	C11—C12—O3—C9	−15.8 (4)
C10—C11—C16—O4	179.2 (3)	O4—C16—O5—C17	1.7 (5)
C12—C11—C16—O5	−178.1 (3)	C11—C16—O5—C17	−177.7 (3)
C10—C11—C16—O5	−1.5 (4)	C18—C17—O5—C16	163.9 (4)
C6A—C1A—C2A—C3A	0.0 (5)	C4A—C5A—O1A—C7A	179.7 (3)
C1A—C2A—C3A—C4A	0.2 (5)	C6A—C5A—O1A—C7A	−0.2 (4)
C2A—C3A—C4A—C5A	−0.5 (5)	O2A—C7A—O1A—C5A	−178.2 (3)
C3A—C4A—C5A—O1A	−179.5 (3)	C8A—C7A—O1A—C5A	2.3 (4)
C3A—C4A—C5A—C6A	0.5 (5)	C8A—C9A—O3A—C12A	12.0 (4)
O1A—C5A—C6A—C1A	179.7 (3)	C6A—C9A—O3A—C12A	−168.6 (2)
C4A—C5A—C6A—C1A	−0.2 (5)	N1A—C12A—O3A—C9A	167.9 (3)
O1A—C5A—C6A—C9A	−0.5 (4)	C11A—C12A—O3A—C9A	−12.6 (4)
C4A—C5A—C6A—C9A	179.6 (3)	O4A—C16A—O5A—C17A	1.5 (5)
C2A—C1A—C6A—C5A	−0.1 (5)	C11A—C16A—O5A—C17A	−179.1 (3)
C2A—C1A—C6A—C9A	−179.8 (3)	C18A—C17A—O5A—C16A	−176.8 (4)
O2A—C7A—C8A—C9A	176.8 (3)	C14—C13—S1—C15	0.8 (2)
O1A—C7A—C8A—C9A	−3.7 (4)	C10—C13—S1—C15	−177.8 (2)
O2A—C7A—C8A—C10A	−3.5 (5)	N2—C15—S1—C13	0.2 (3)
O1A—C7A—C8A—C10A	176.0 (2)	N2A—C14A—S1A—C13A	0.9 (2)
C7A—C8A—C9A—O3A	−177.4 (3)	C15A—C13A—S1A—C14A	−0.6 (2)
C10A—C8A—C9A—O3A	2.9 (5)	C10A—C13A—S1A—C14A	178.8 (2)

### Hydrogen-bond geometry (Å, º)

**Table:**

*D*—H···*A*	*D*—H	H···*A*	*D*···*A*	*D*—H···*A*
N1—H1*B*···O4	0.86	2.11	2.698 (3)	125
N1—H1*B*···N2*A*^i^	0.86	2.49	3.152 (3)	135
N1—H1*C*···N2^ii^	0.86	2.27	3.116 (4)	167
N1*A*—H1*A*2···N2*A*^iii^	0.86	2.13	2.988 (4)	174
N1*A*—H1*A*1···O4*A*	0.86	2.09	2.690 (3)	126
N1*A*—H1*A*1···O4^ii^	0.86	2.52	2.936 (3)	111

Symmetry codes: (i) *x*, *y*, *z*+1; (ii) −*x*+1/2, *y*+1/2, −*z*+3/2; (iii) −*x*+1/2, *y*+1/2, −*z*+1/2.

## References

[bb1] Abd-El-Aziz, A. S., El-Agrody, A. M., Bedair, A. H., Corkery, T. C. & Ata, A. (2004). *Heterocycles*, **63**, 1793–1812.

[bb2] Alvey, L., Prado, S., Huteau, V., Saint-Joanis, B., Michel, S., Koch, M., Cole, S. T., Tillequin, F. & Janin, Y. L. (2008). *Bioorg. Med. Chem.* **16**, 8264–8272. 10.1016/j.bmc.2008.06.057 18752967

[bb3] Alvey, L., Prado, S., Saint-Joanis, B., Michel, S., Koch, M., Cole, S. T., Tillequin, F. & Janin, Y. L. (2009). *Eur. J. Med. Chem.* **44**, 2497–2505. 10.1016/j.ejmech.2009.01.017 19232450

[bb4] Bedair, A. H., Emam, H. A., El-Hady, N. A., Ahmed, K. A. R. & El-Agrody, A. M. (2001). *Il Farmaco*, **56**, 965–973. 10.1016/s0014-827x(01)01168-5 11829118

[bb5] Bruker (2004). *APEX2*, *SAINT-Plus* and *SADABS* Bruker AXS Inc., Madison, Wisconsin, USA.

[bb6] El-Agrody, A. M., Eid, F. A., Emam, H. A., Mohamed, H. M. & Bedair, A. H. (2002). *Z. Naturforsch. Teil B*, **57**, 579–585.

[bb7] El-Agrody, A. M., Sabry, N. M. & Motlaq, S. S. (2011). *J. Chem. Res.* **35**, 77–83.

[bb8] Farrugia, L. J. (2012). *J. Appl. Cryst.* **45**, 849–854.

[bb9] Jeso, V. & Nicolaou, K. C. (2009). *Tetrahedron Lett.* **50**, 1161–1163. 10.1016/j.tetlet.2008.12.096 PMC2683023 20161287

[bb10] Sabry, N. M., Mohamed, H. M., Khattab, E. S. A. E. H., Motlaq, S. S. & El-Agrody, A. M. (2011). *Eur. J. Med. Chem.* **46**, 765–772. 10.1016/j.ejmech.2010.12.015 21216502

[bb11] Sheldrick, G. M. (2008). *Acta Cryst.* A**64**, 112–122. 10.1107/S0108767307043930 18156677

[bb12] Shi, D.-Q., Wang, J., Wang, X., Zhuang, Q. & Yu, K. (2003). *Acta Cryst.* E**59**, o1733–o1734.

[bb13] Wang, J., Shi, D. & Wang, X. (2004). *Acta Cryst.* E**60**, o1725–o1727.

